# Brain Activation Patterns at Exhaustion in Rats That Differ in Inherent Exercise Capacity

**DOI:** 10.1371/journal.pone.0045415

**Published:** 2012-09-17

**Authors:** Teresa E. Foley, Leah R. Brooks, Lori J. Gilligan, Paul R. Burghardt, Lauren G. Koch, Steven L. Britton, Monika Fleshner

**Affiliations:** 1 Department of Integrative Physiology and The Center for Neuroscience, University of Colorado, Boulder, Colorado, United States of America; 2 Department of Anesthesiology, University of Michigan, Ann Arbor, Michigan, United States of America; 3 Department of Psychiatry and the Molecular and Behavioral Neuroscience Institute, University of Michigan, Ann Arbor, Michigan, United States of America; University of Las Palmas de Gran Canaria, Spain

## Abstract

In order to further understand the genetic basis for variation in inherent (untrained) exercise capacity, we examined the brains of 32 male rats selectively bred for high or low running capacity (HCR and LCR, respectively). The aim was to characterize the activation patterns of brain regions potentially involved in differences in inherent running capacity between HCR and LCR. Using quantitative *in situ* hybridization techniques, we measured messenger ribonuclease (mRNA) levels of c-Fos, a marker of neuronal activation, in the brains of HCR and LCR rats after a single bout of acute treadmill running (7.5–15 minutes, 15° slope, 10 m/min) or after treadmill running to exhaustion (15–51 minutes, 15° slope, initial velocity 10 m/min). During verification of trait differences, HCR rats ran six times farther and three times longer prior to exhaustion than LCR rats. Running to exhaustion significantly increased c-Fos mRNA activation of several brain areas in HCR, but LCR failed to show significant elevations of c-Fos mRNA at exhaustion in the majority of areas examined compared to acutely run controls. Results from these studies suggest that there are differences in central c-Fos mRNA expression, and potential brain activation patterns, between HCR and LCR rats during treadmill running to exhaustion and these differences could be involved in the variation in inherent running capacity between lines.

## Introduction

In the last few decades, exercise physiologists have turned to animal models to supplement the ongoing research on the genetic basis for differences in inherent (untrained) exercise capacity in humans. By controling the environmental conditions with which animals are housed, researchers are better able to isolate potential “exercise genes” that may contribute to differences in inherent running capacity. To date, most of the research in exercise genomics in animals has focused on cardiac and skeletal muscle adaptations [1], [2], [3], [4], [5], [6], [7], [8], [9], [10], [11], [12], [13], [14], [15], [16], [17], [18], while the contribution of central mechanisms remains less understood. Because the initiation and processing of locomotor activity occurs within the central nervous system, we hypothesized that alterations in central pathways could also play a role in variation in inherent running capacity between animals.

In 1996, a large-scale selective breeding program in rats was initiated to create divergent lines of inherent running capacity to serve as substrate to help understand the genetic basis for the variation in maximal running capacity [9], [19]. For development of these models, running capacity was assessed at each generation by using an incremental velocity treadmill running protocol to exhaustion when the animals were 11 weeks of age [20]. After 6 generations of selection, the low capacity runners (LCR) and high capacity runners (HCR) differed in inherent running capacity by 171%, with the HCR diverging from the founding line more than the LCR [20]. Selection simultaneously produced contrasts between the lines for numerous physiological variables including body weight [9], [20], cardiovascular function [9], [10], [11], [12], [13], lipid and glucose metabolism [21], [22], [23], [24], maximal oxygen consumption [25], [26], [27], muscle and liver oxidative capacity [26], [28], [29], [30], mitochondrial function [9], [31], [32], [33], nociception [34], [35], endocrine stress responsiveness [36], [37], longevity [38], and spontaneous wheel running activity [36], [39], [40] to name a few.

The aim of the current experiment was to identify brain regions potentially involved in the variation in inherent running capacity between HCR and LCR rats from generation 19 during treadmill running to exhaustion. Although Rhodes et al. (2003) have defined markers of neuronal brain activity that differ between control line mice and mice selectively bred for increased voluntary wheel running activity [41], these experiments did not evaluate an end-point of exhaustion. Thus, we examined 25 brain areas of HCR and LCR rats for the expression of the immediate early gene c-Fos at multiple time points after running onset and after an exhaustive run (see Table 1). Several of these areas have been shown in previous studies to induce c-Fos expression after treadmill running including the hippocampus [42], [43], [44], [45], [46], paraventricular nucleus of the hypothalamus (PVN) [47], [48], [49] arcuate nucleus (Arc) [47], cortex [48], medulla [50], caudate putamen (CPU) [48], [51], striatum [52], lateral septal nucleus (LS) [48], pontine nucleus (Pn) [48], locus coeruleus (LC) [48], cingulate cortex (Cg1) [48], somatosensory cortex (S1) [48], and periaqueductal gray (PAG) [53].

**Table 1:** Brain region, abbreviation, and bregma coordinates of brain areas quantified in high capacity runners (HCR) and low capacity runners (LCR).

Brain Region	Abbreviation	Bregma Coordinates
1^st^ Cerebellar lobe	1Cb	−9.72 to −10.80 mm posterior
Arcuate Nucleus	Arc	−1.80 to −3.24 mm posterior
CA field of Hippocampus	CA1-3	−2.64 to −5.28 mm posterior
Caudate Putamen	CPu	2.28 mm ant. to −0.60 mm post.
Cingulate Cortex	Cg1	3.24 to 0.24 mm anterior
Dentate Gyrus	DG	−2.64 to −5.28 mm posterior
Entorhinal Cortex	Ent	−6.24 to −8.52 mm posterior
Inferior Olivary Nucleus	OLV	−9.12 to −10.20 mm posterior
Infralimbic Cortex	IL	3.72 to 2.52 mm anterior
Locus Coeruleus	LC	−9.48 to −10.20 mm posterior
Lateral Septal Nucleus	LS	1.68 to 0.60 mm anterior
Median Eminence	ME	−1.80 to −3.24 mm posterior
Nucleus Accumbens	Acb	2.76 to 0.84 mm anterior
Paraventricular Nucleus	PVN	−1.08 to −2.04 mm posterior
Periaqueductal Gray	PAG	−6.00 to −8.40 mm posterior
Piriform Cortex	Pir	3.24 to 0.12 mm anterior
Pontine Nuclei	Pn	−6.84 to −7.92 mm posterior
Prelimbic Cortex	PrL	3.72 to 2.52 mm anterior
Primary Motor Cortex	M1	3.24 to 0.24 mm anterior
Primary Somatosensory Cortex	S1	3.24 to 0.24 mm anterior
Raphe Magnus Nucleus	RMg	−9.72 to −12.00 mm posterior
Raphe Pallidus Nucleus	RPa	−9.72 to −12.00 mm posterior
Secondary Motor Cortex	M2	3.24 to 0.24 mm anterior

Bregma coordinates according to [84].

## Results

### 1. Body weight and treadmill tests at weeks 11 and 30

As part of standard protocol, treadmill tests were performed at week 11 to verify trait differences in inherent exercise capacity between HCR and LCR. Consistent with previous reports [9], [20], HCR weighed significantly less and ran significantly farther and longer than LCR. The average body weights for HCR and LCR at week 11 were 201±4 and 343±6 grams, respectively [F (1,30) = 387; p<0.0001]. In terms of distance run, HCR ran a maximum distance of 1998±28 meters, whereas LCR ran a maximum distance of 212±6 meters (943% difference; [F (1,30) = 4026; p<0.0001] In addition, HCR ran for a maximum duration of 72±0.60 minutes while LCR ran for a maximum duration of 15±0.35 minutes [F (1,30) = 6743; p<0.0001]).

**Figure 1:**
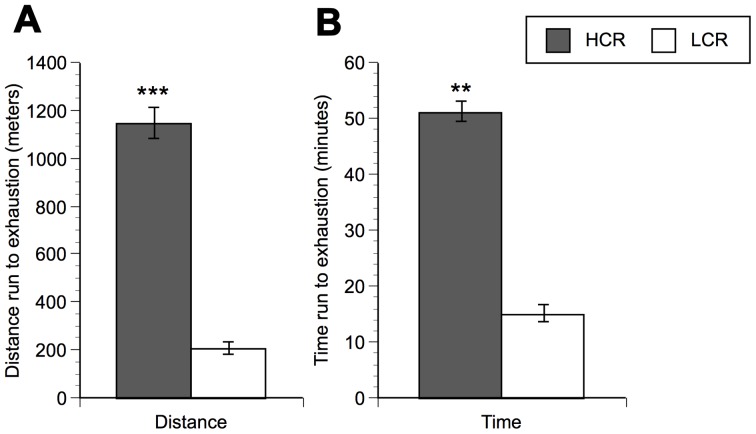
Treadmill tests in high and low capacity runners at week 30. Panel A represents distance run to exhaustion between HCR and LCR. Panel B represents time run to exhaustion between HCR and LCR. N = 8 animals/group. Values represent group means ± standard error of measurement. Fisher protected least significant difference: ** p<.01, *** p<.0001 with respect to LCR at respective time point.

These patterns were consistent with the second set of treadmill tests performed at week 30. By week 30, LCR were still significantly heavier than HCR. HCR weighed 294±8 grams, whereas LCR weighed 521±11 grams [F (1,30) = 301; p<0.0001]. At this time point, HCR ran a maximum distance of 1149±66 meters, whereas LCR ran a maximum distance of 205±26 meters (562% difference; [F (3,28) = 197; p<0.0001]). Similar to week 11, HCR ran significantly longer to exhaustion compared to LCR at week 30. HCR ran a maximum duration of 51±2 minutes while LCR ran a maximum duration of 15±1.5 minutes (338% difference; [F (3,28) = 278; p<0.0001]). Figure 1A represents the maximum distance run (in meters) and the corresponding time (in minutes) recorded during the best out of five runs to exhaustion at week 30. Figure 1B shows the same pattern for time to exhaustion between groups at week 30.

### 2. Relative c-Fos mRNA levels across groups in 25 brain areas

**Figure 2:**
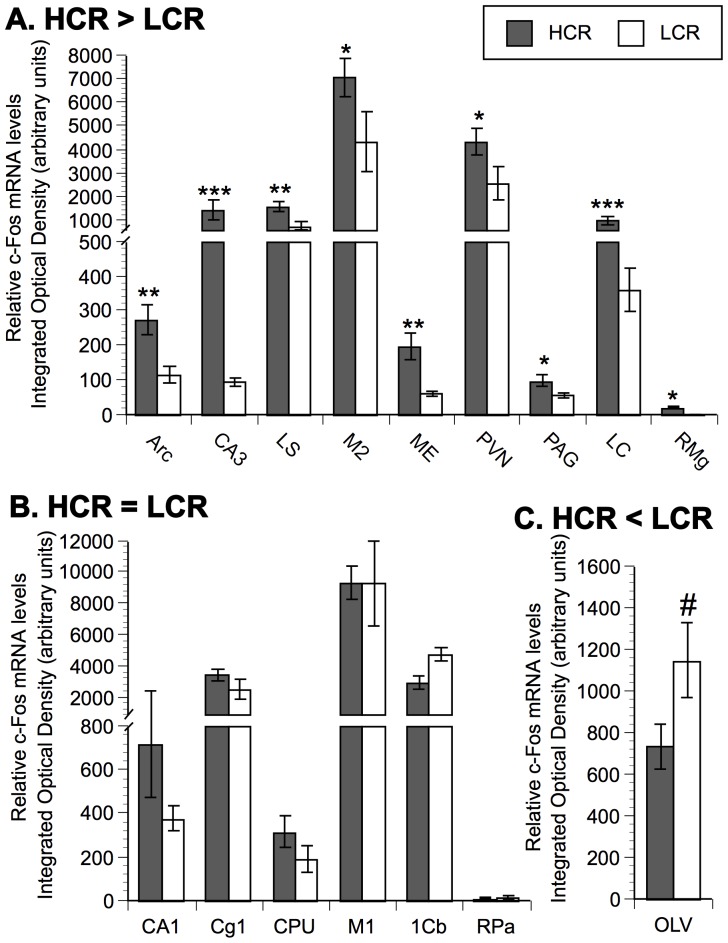
Comparison of relative c-Fos mRNA levels between HCR and LCR run to exhaustion. Panel A represents brain areas where c-Fos mRNA is statistically higher in HCR versus LCR (HCR>LCR). Panel B represents brain areas where there is no statistical difference in mRNA expression between HCR and LCR (HCR = LCR). Panel C represents brain areas where c-Fos mRNA is statistically lower in HCR versus LCR (HCR<LCR). N = 8 animals/group. Values represent group means ± standard error of measurement. Fisher protected least significant difference: * p<.05, ** p<.01, *** p<.001 with respect to LCR; # p<.05 with respect to HCR.

In the current study, 16 of the 25 brain areas examined demonstrated significant differences in c-Fos mRNA expression across groups based on one-way ANOVA analyses. Within these brain areas, F-PLSD post hoc analyses were performed and the individual post hoc results are reported below and presented in Figures 2–5. C-Fos mRNA levels of the nine brain areas not significantly different between groups are represented in Table 2.

**Figure 3 pone-0045415-g003:**
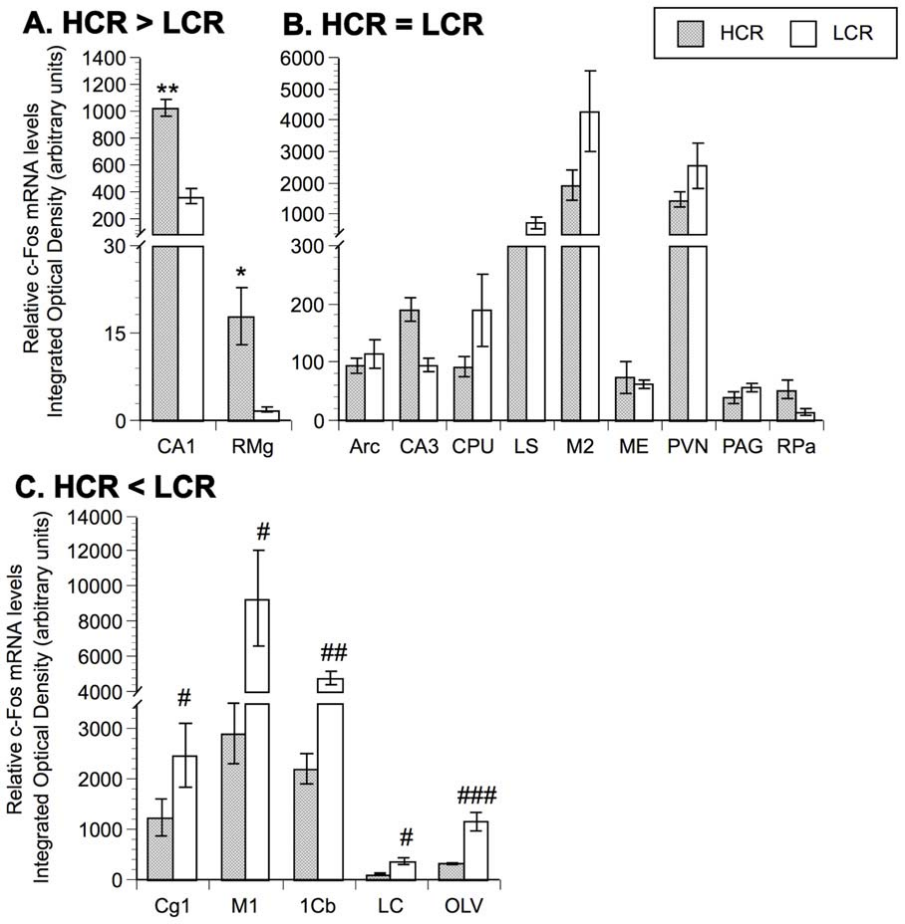
Comparison of relative c-Fos mRNA levels between HCR and LCR run for 15 minutes. Panel A represents brain areas where c-Fos mRNA is statistically higher in HCR versus LCR (HCR>LCR). Panel B represents brain areas where there is no statistical difference in mRNA expression between HCR and LCR (HCR = LCR). Panel C represents brain areas where c-Fos mRNA is statistically lower in HCR versus LCR (HCR<LCR). N = 8 animals/group. Values represent group means ± standard error of measurement. Fisher protected least significant difference: * p<.05, ** p<.01 with respect to LCR; # p<.05, ## p<.01, ### p<.001 with respect to HCR.

**Figure 4 pone-0045415-g004:**
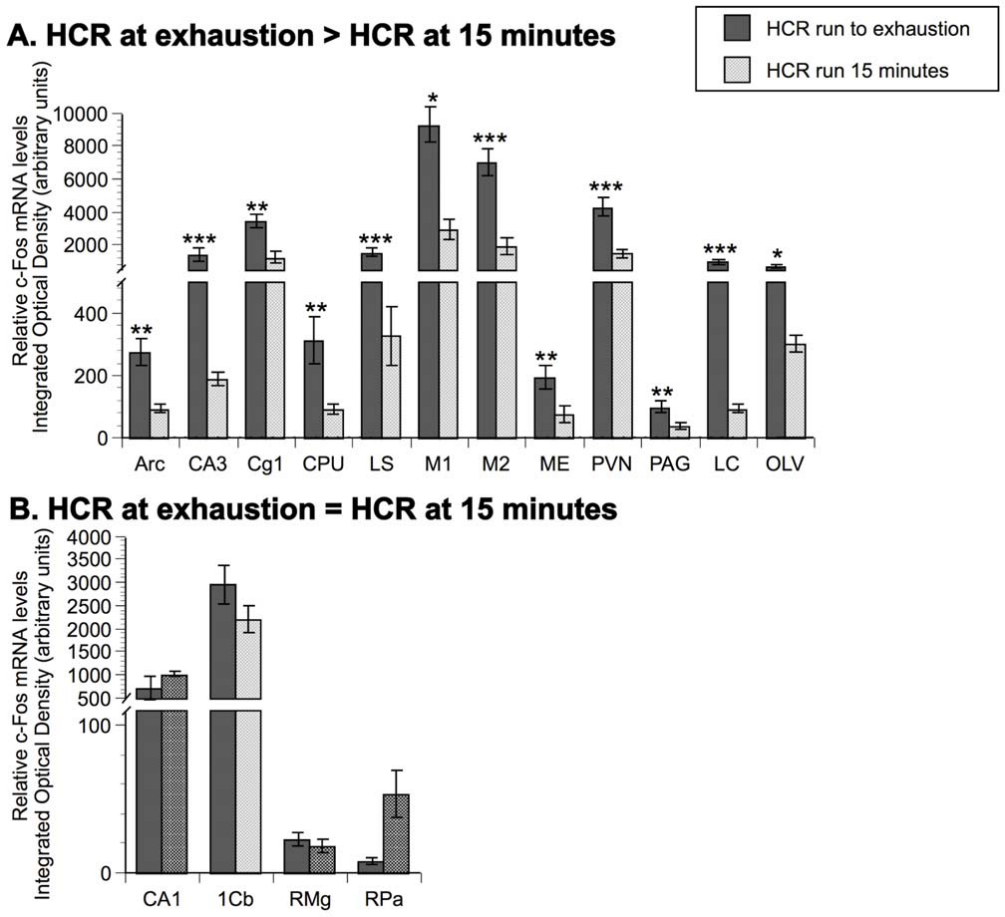
Comparison of relative c-Fos mRNA levels in HCR. Panel A represents brain areas where c-Fos mRNA is statistically higher in HCR run to exhaustion versus HCR after 15 minutes of running (HCR at exhaustion>HCR at 15 minutes). Panel B represents brain areas where there is no statistical difference in mRNA expression between HCR at different time points (HCR at exhaustion  = HCR at 15 minutes). N = 8 animals/group. Values represent group means ± standard error of measurement. Fisher protected least significant difference: * p<.05, ** p<.01, *** p<.001 with respect to HCR run for 15 minutes.

**Figure 5:**
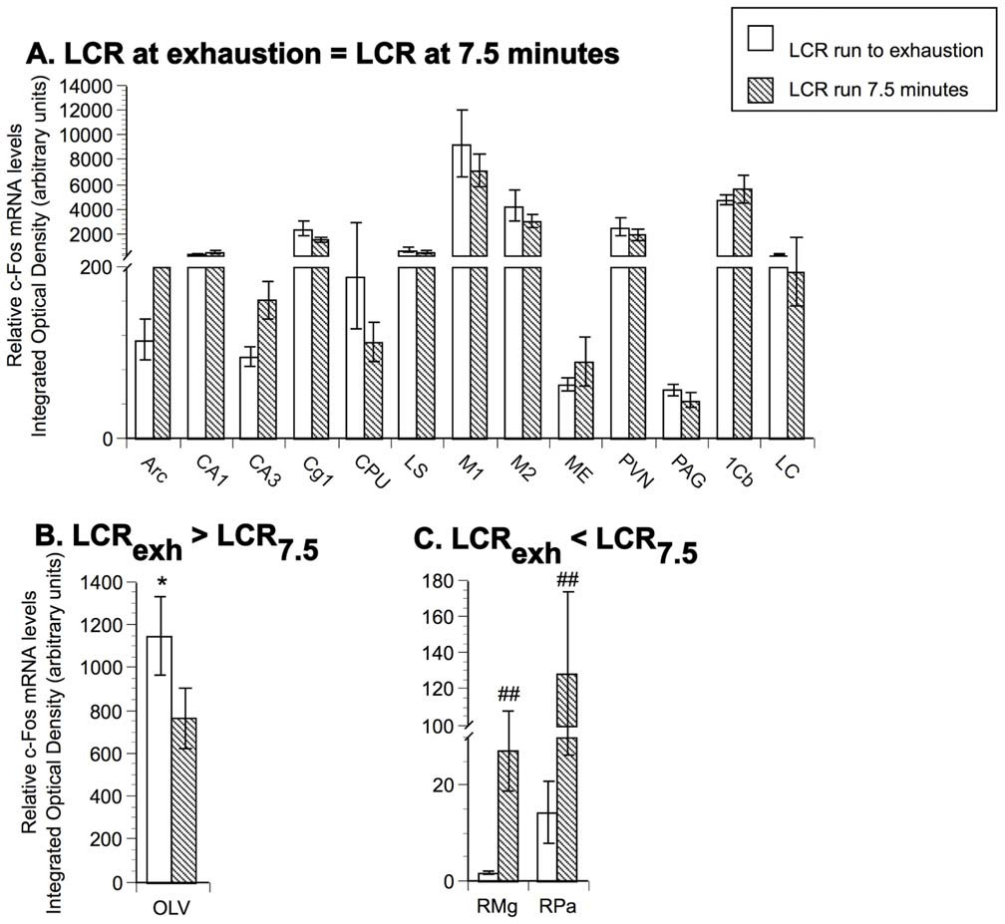
Comparison of relative c-Fos mRNA levels in LCR. Panel A represents brain areas where there is no statistical difference in mRNA expression between LCR at different time points (LCR at exhaustion  = LCR at 7.5 minutes). Panel B represents brain areas where c-Fos mRNA is statistically higher in LCR run to exhaustion versus LCR after 7.5 minutes of running (LCR_exh_>LCR_7.5_). Panel C represents brain areas where c-Fos mRNA is statistically lower in LCR run to exhaustion versus LCR after 7.5 minutes of running (LCR_exh_<LCR_7.5_). N = 8 animals/group. Fisher protected least significant difference: * p<.05 with respect to LCR run 7.5 minutes; ## p<.01 with respect with LCR run to exhaustion.

**Table 2:** C-Fos mRNA expression in HCR and LCR in response to treadmill running.

	HCR		LCR			
Brain Region	Run 15 minutes (n = 8)	Exhaustion (n = 8)	Run 7.5 minutes (n = 8)	Exhaustion (n = 8)	F value (3, 28)	P value
Acb	74±18	139±23	123±19	93±22	0.1	0.14
CA2	115±4	116±38	62±11	52±7	2.8	0.06
DG	75±14	147±47	86±24	61±8	1.9	0.16
IL	894±271	2451±535	1687±345	2126±847	1.5	0.23
Pir	4774±780	6109±671	7520±744	7436±1583	1.6	0.20
PrL	1245±311	4666±1180	2352±418	3340±1193	2.7	0.06
S1	3497±895	7779±886	6533±974	9870±2720	2.9	0.055
Ent	361±124	653±226	458±95	449±106	0.7	0.58
Pn	1294±328	1326±275	971±151	873±125	0.9	0.44

Values represent average densities ± standard error of the mean. There was no significant difference in c-Fos mRNA expression across groups in nine of the 25 brain areas examined (shown here).

### 3. Comparison of relative c-Fos mRNA levels between HCR and LCR run to exhaustion

Brain c-Fos mRNA levels were assessed in HCR and LCR groups run to exhaustion. As indicated in Figure 2A, HCR had significantly higher c-Fos mRNA levels than LCR at exhaustion in nine of the brain regions examined (Arc, CA3, LS, M2, ME, PVN, PAG, LC, RMg). There was no significant difference in c-Fos mRNA between lines in six brain areas (CA1, Cg1, CPU, M1, 1Cb, RPa) as indicated in Figure 2B, while LCR demonstrated higher c-Fos mRNA levels than HCR at exhaustion in only one brain area (OLV) as indicated in Figure 2C.

### 4. Comparison of relative c-Fos mRNA levels between HCR and LCR run for 15 minutes

In order to compare the brain areas activated by an equal duration of treadmill running between lines, c-Fos mRNA levels were assessed in HCR and LCR after 15 minutes of running. It should be kept in mind, however, that after 15 minutes of running LCR rats had reached exhaustion (Fig. 1), while the HCR rats were still exercising. After 15 minutes of running, HCR demonstrated higher levels of c-Fos mRNA in two brain areas (CA1, RMg) compared to LCR (Fig. 3A). There was no significant difference in c-Fos mRNA in nine brain regions examined (Arc, CA3, CPU, LS, M2, ME, PVN, PAG, RPa) as indicated in Figure 3B, while LCR demonstrated higher c-Fos mRNA levels in five brain areas (Cg1, M1, 1Cb, LC, OLV) after 15 minutes of running compared to HCR (Fig. 3C).

### 5. Comparison of relative c-Fos mRNA levels between animals run to exhaustion and acute run controls

Figure 4 shows the comparison of c-Fos mRNA levels between HCR run to exhaustion and HCR during 15 minutes of running (acute run controls). HCR run to exhaustion had significantly more c-Fos mRNA in 12 brain regions (Arc, CA3, Cg1, CPU, LS, M1, M2, ME, PVN, PAG, LC, OLV) compared to HCR after 15 minutes of running (Fig. 4A). There was no significant difference in c-Fos mRNA between HCR groups in four brain areas (CA1, 1Cb, RMg, RPa) as indicated by Figure 4B.

In contrast, Figure 5A shows that there was no significant difference in c-Fos mRNA between LCR run to exhaustion and LCR during 7.5 minutes of running (acute run controls) in 13 of the brain areas examined (Arc, CA1, CA3, Cg1, CPU, LS, M1, M2, ME, PVN, PAG, 1Cb, LC). Interestingly, LCR run to exhaustion had significantly more c-Fos mRNA in the OLV compared to LCR after 7.5 minutes of running (Fig. 5B). Alternatively, LCR that ran only 7.5 minutes had significantly more c-Fos mRNA in the RMg and RPa compared to LCR run to exhaustion (Fig. 5C).

### 6. Correlations between c-Fos mRNA levels and distance run during treadmill test at week 30

Significant positive correlations (R>0.5) were found between the levels of c-Fos mRNA and distance run during the treadmill tests in HCR rats in 15 of the brain areas examined: Acb, Arc, CA3, Cg1, CPU, IL, LS, M1, M2, PrL, PVN, S1, PAG, LC, OLV. A significant negative correlation was found between c-Fos mRNA levels and distance run in HCR in the RPa. In LCR rats, a significant positive correlation for c-Fos mRNA and distance run was found in the IL and PrL. See Table 3 for p-values and correlation coefficients between distance run and c-Fos mRNA levels.

**Table 3:** Correlation between relative c-Fos mRNA and distance run during treadmill tests at week 30 of HCR and LCR.

	HCR		LCR	
Brain Region	Correlation coefficient (R)	P-value	Correlation coefficient (R)	P-value
Acb	0.542	0.03*	−0.001	0.99
Arc	0.724	0.0009***	−0.279	0.30
CA1	−0.337	0.21	−0.287	0.28
CA2	−0.092	0.74	−0.121	0.66
CA3	0.533	0.03*	−0.422	0.10
Cg1	0.716	0.001***	0.279	0.30
CPu	0.600	0.01**	0.280	0.30
DG	0.380	0.15	−0.193	0.48
IL	0.649	0.005**	0.489	0.05*
LS	0.827	<0.0001***	0.430	0.10
M1	0.810	<0.0001***	0.121	0.66
M2	0.844	<0.0001***	0.216	0.43
ME	0.463	0.07	−0.194	0.48
Pir	0.426	0.10	0.399	0.13
PrL	0.732	0.0008***	0.501	0.05*
PVN	0.776	0.0002***	0.055	0.84
S1	0.664	0.004**	0.252	0.35
Ent	−0.114	0.71	0.482	0.07
PAG	0.553	0.02	0.094	0.73
Pn	0.005	0.99	−0.411	0.12
1Cb	0.258	0.34	−0.262	0.33
LC	0.840	<0.0001***	−0.411	0.12
OLV	0.735	0.0007***	0.346	0.19
RMg	0.203	0.46	−0.486	0.06
RPa	−0.580	0.02*	−0.436	0.09

Fisher protected least significant difference: * p<.05, ** p<.01, *** p<.001.

### 7. Plasma corticosterone levels in HCR and LCR after varying durations of treadmill running

**Figure 6:**
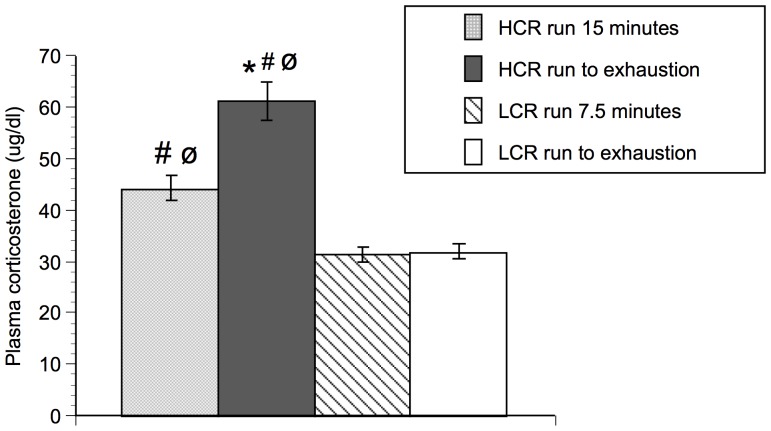
Plasma corticosterone levels in HCR and LCR after varying durations of treadmill running. N = 8 animals/group. Values represent group means ± standard error of measurement. Fisher protected least significant difference: * p<0.001 with respect to HCR run to 15 minutes; # p<0.001 with respect to LCR run to exhaustion; ø p<0.001 with respect to LCR run 7.5 minutes.

Figure 6 represents the plasma corticosterone levels obtained in HCR and LCR rats at week 30. One-way ANOVA revealed a reliable main effect of group for plasma corticosterone levels [F (3,28) = 33; p<0.0001]. F-PLSD post hoc analysis revealed that HCR run to exhaustion had significantly higher plasma corticosterone levels than HCR run 15 minutes (p<0.0001), LCR run to exhaustion (p<0.0001), and LCR run 7.5 minutes (p<0.0001). Furthermore, HCR run 15 minutes had significantly higher plasma corticosterone levels than LCR run to exhaustion (F-PLSD, p = 0.0008) and LCR run 7.5 minutes (F-PLSD, p = 0.001). There was no difference in plasma corticosterone levels between LCR run to exhaustion and LCR run 7.5 minutes.

## Discussion

The aim of the current experiment was to characterize the activation patterns of brain regions potentially involved in differences in inherent (untrained) running capacity between HCR and LCR. Using c-Fos mRNA as a marker of neuronal activation, we examined the brains of HCR and LCR rats during a single bout of acute treadmill running or during treadmill running to exhaustion. During the treadmill testing at week 30, HCR rats ran 562% farther and 338% longer to exhaustion than LCR rats. Running to exhaustion significantly increased c-Fos mRNA activation of several brain areas in HCR, but LCR failed to show significant elevations of c-Fos mRNA in the majority of areas examined at exhaustion compared to acutely run controls. Results from these studies suggest that there are differences in central c-Fos mRNA expression, and possibly brain activation patterns, between HCR and LCR rats during treadmill running to exhaustion, and these differences may contribute to the variation in inherent running capacity between lines.

In both HCR and LCR, running to exhaustion significantly increased activation of certain brain areas involved in motor control and motivation. These areas include the CPU (initiation of movement; [54], [55]), M1 (execution of movement; [56]) 1Cb (postural control; [57], [58]), and Cg1 (anticipation of tasks and motivation; [59]). However, because these areas were stimulated at exhaustion in both lines, this suggests that activation of these regions may not contribute to differences in inherent running capacity. Rather, it is likely that activation of other brain areas may be responsible for the differences in inherent running capacity between lines. We propose that areas involved in pain analgesia and stress/arousal may be involved in the mechanisms by which HCR have enhanced inherent running capacity compared to LCR.

### 1. Potential mechanisms by which HCR have enhanced inherent running capacity

Previous behavioral data has shown that HCR animals have higher pain thresholds compared to LCR animals both before and after exercise to exhaustion [34]. Based on these observations, it is possible that activation of areas involved in pain analgesia, such as the PAG [60], RMg [61], and LC [62] may be involved in the mechanisms by which HCR have enhanced inherent running capacity than LCR. The PAG sends excitatory projections to serotonergic neurons in the RMg [62]. When activated, these areas produce analgesia through inhibition of nociceptive neurons in the dorsal horn of the spinal cord [62]. These nociceptive neurons are also inhibited by noradrenergic neurons in the LC [62], [63]. Consequently, it is possible that HCR are better able than LCR to activate these areas to reduce the sensation of pain and elevate pain thresholds during treadmill running to exhaustion. Consistent with our hypothesis, HCR demonstrated higher c-Fos mRNA levels in the PAG, RMg, and LC at exhaustion than LCR. Additionally, a positive correlation (R = 0.840) was found between c-Fos mRNA levels in the LC and distance run in HCR at week 30.

In addition to pain analgesic systems, it is possible that activation of stress and arousal circuits could also contribute to the enhanced inherent running capacity observed in HCR rats. Activation of the hypothalamic-pituitary-adrenal (HPA) axis by stress or physical exertion ultimately leads to the release of the hormone corticosterone by the adrenal cortex. Once released, corticosterone increases the mobilization of fat and glycogen stores during exercise [64], [65], [66]. Because HCR ran a maximum distance of ∼1150 meters for 51 minutes during testing, it is likely that the physiological demand of treadmill running to exhaustion was sufficient to activate the HPA axis and stimulate plasma corticosterone release in HCR rats. Indeed, previous studies have shown increases in plasma corticosterone after 25 or 40 minutes of treadmill running in male and female mice, respectively [67].

As hypothesized, HCR run to exhaustion had significant elevations in c-Fos mRNA expression in the PVN and plasma corticosterone levels compared to LCR rats and acutely run HCR controls, suggesting that the HPA axis in these rats is indeed activated. Furthermore, given that the HPA axis is subject to modulation by a variety of brain areas, an increase in activation of afferents to the PVN could also stimulate the release of corticosterone and enhance inherent running capacity in HCR. Consistent with this idea, HCR also demonstrated higher c-Fos mRNA expression in the LC and LS, two major afferents of the PVN [68], [69] at exhaustion than LCR. When compared to distance run at week 30, statistical analyses revealed a positive correlation to c-Fos mRNA within the PVN (R = 0.776), LC (0.840), and LS (0.827) of HCR animals. Collectively, these data suggest that exercise-induced stimulation of stress and arousal circuits and corticosterone release may be involved in the mechanisms by which HCR and LCR differ in inherent running capacity.

The increased activation of stress and arousal circuits in HCR rats might also suggest that HCR rats find treadmill running to exhaustion more psychologically stressful than their LCR counterparts. We argue against this scenario as previous behavioral data in these animals has demonstrated that HCR rats exhibit less anxiety-like behavior in response to restraint stress [37], contextually conditioned cat odor [70], forced swim testing [70], and novel environments [37] compared to their LCR counterparts. Furthermore, HCR express increased plasma corticosterone levels compared to LCR during several of these behavioral tests [37], [70]. Given that HCR are behaviorally less anxious than LCR in the absence of specific danger signals, these data suggest that it is unlikely that HCR rats find treadmill running to exhaustion more psychologically stressful than LCR rats. Rather, the increased activation of stress and arousal circuits and plasma corticosterone release in HCR may be a response to the physiological demand of treadmill running.

We also analyzed relative levels of c-Fos mRNA expression between HCR run to exhaustion and their acutely run treadmill controls. Running to exhaustion significantly elevated activation of several brain areas in HCR compared to acutely run controls. These areas included those involved in motor control (CPU, M1, M2), analgesia (PAG, LC), and stress and arousal circuits (PVN, LC, LS), further confirming our hypothesis that activation of these areas may be involved in the mechanisms by which HCR have enhanced inherent running capacity during treadmill running to exhaustion.

Because HCR ran six times longer and three times farther to exhaustion than LCR prior to sacrifice, one could argue that the increase in c-Fos mRNA expression observed in HCR rats was due to enhanced stimulation of these brain areas produced by treadmill running to exhaustion by itself, and was not due to differences in brain activation patterns produced by artificial selection. Indeed, the dynamics of c-Fos mRNA expression are sensitive to the duration of stimulation [71], [72], [73]. However, this scenario is unlikely for several reasons. First, not all brain areas examined in HCR showed greater c-Fos mRNA expression at exhaustion than LCR. In fact, there was similar c-Fos mRNA expression between lines at exhaustion in six brain areas, and reduced c-Fos mRNA expression in one area of HCR, compared to LCR. Second, there were still differences in c-Fos mRNA between time-matched HCR and LCR, suggesting that the differences in c-Fos mRNA expression are not due to the duration of treadmill running. Although the time course of c-Fos mRNA expression has not been directly examined during treadmill running to exhaustion, it is possible that the c-Fos mRNA values reported in HCR may be decreasing due to the longer duration of treadmill running. Therefore, it is more likely that the differences in c-Fos mRNA expression represent the genetic differences in brain activation patterns between these lines, and not simply differences in time spent running.

### 2. Potential mechanisms by which LCR have reduced inherent running capacity

In addition to the potential mechanisms by which HCR have enhanced inherent running capacity, here we propose several different mechanisms by which LCR have *reduced* inherent running capacity compared to HCR. When examining c-Fos mRNA expression between animals run to exhaustion and their acutely run treadmill controls, LCR show very little change in c-Fos mRNA across brain areas compared to HCR. The lack of major difference in c-Fos mRNA levels, and potential brain activation patterns, in LCR implies that exercise capacity limiting systems are different in LCR than HCR when treadmill running to exhaustion.

We propose that activation of the OLV may be one brain area responsible for the reduced inherent running capacity observed in LCR. The OLV is a major afferent of the cerebellum that coordinates the temporal firing of muscles during movement [74] and has been implicated in the exercise pressor reflex [75]. When initiated, the exercise pressure reflex initiates increases in heart rate, blood pressure, and ventilation [76], [77], [78].We propose that over-stimulation of the OLV in LCR might impair the exercise pressure reflex, thereby limiting performance during running. Compared to HCR rats and acutely run LCR controls, LCR run to exhaustion had significant elevations in c-Fos mRNA expression in the OLV, suggesting that activation of this area might be involved in the mechanisms by which LCR have reduced inherent running capacity compared to HCR. Future studies are required to further understand the role of the OLV in the exercise pressure reflex in LCR animals.

It is also possible that a reduction in activation of serotonergic neurons in the RMg or RPa may be involved in the reduced inherent running capacity in LCR. As stated earlier, activation of serotonergic neurons in the RMg produces analgesia through inhibition of nociceptive neurons in the dorsal horn of the spinal cord [62]. Reduced activation of the RMg by LCR would reduce inhibition of the nociceptive neurons in the dorsal horn, thereby increasing the sensation of pain during treadmill running to exhaustion. Furthermore, reduced activation of serotonergic neurons in the RPa during treadmill running has been linked with central fatigue in awake cats [79]. Compared to HCR and acutely run LCR controls, LCR rats run to exhaustion demonstrated reduced c-Fos mRNA expression in the RMg and RPa, suggesting that a reduced activation of the RMg and RPa may reduce inherent running capacity in LCR.

The lack of major difference in relative c-Fos mRNA levels between LCR run to exhaustion and their acute run controls also implies that central mechanisms may not be the limiting factor in LCR rats during treadmill running to exhaustion. Rather, it is possible that peripheral factors in LCR may account for the reduced inherent running capacity observed in LCR rats. In addition to being heavier than their HCR counterparts, LCR rats score high on several cardiovascular risk factors associated with the metabolic syndrome such as elevated blood pressure, insulin resistance, and higher visceral adiposity, plasma triglycerides, and plasma free fatty acids [9], which may impair their ability to run. Additionally, LCR demonstrate lower maximal oxygen consumption (VO_2max_) [9], [25], [26], [29], reduced skeletal muscle oxygen conductance [26], [29], and lower total muscle fiber number [26], [29] than their HCR counterparts. When taken together, it would appear that the underlying mechanisms regulating treadmill running to exhaustion are multifaceted and distinct among HCR and LCR rats.

### 3. Conclusion

These results help to identify potential brain areas involved in the variation in inherent (untrained) running capacity between HCR and LCR during treadmill running to exhaustion. HCR animals demonstrate increased c-Fos mRNA activation of central pain analgesic and stress/arousal circuits during treadmill running to exhaustion, which may contribute to their enhanced inherent running capacity, compared to LCR animals. In contrast, LCR rats may have lower inherent running capacity than their HCR counterparts due to over-activation of c-Fos mRNA in the OLV and/or reduced c-Fos mRNA in caudal serotonergic systems. This study demonstrates a central architecture that can explain at least part of the difference for exhaustion between HCR and LCR.

## Materials and Methods

### 1. Animals

The development of LCR and HCR rat-lines has been described previously [20]. Briefly, two-way selection was used to create high and low strains of inherent running capacity. The founder population was 168 genetically heterogeneous rats (N:NIH stock) obtained from a colony once maintained at the National Institutes of Health [80]. The 13 lowest- and the 13 highest-capacity rats of each sex were selected from the founder population and randomly paired for mating. Each subsequent generation was stratified and bred in a similar fashion with precaution being taken to minimize inbreeding (<1%/generation). Variation from sampling errors and genetic drift was reduced by an increase in the number of animals maintained at each generation in each selected line. Sixteen HCR and 16 LCR adult males derived from generation 19 were used in the present study.

All animal procedures were carried out in strict accordance with the recommendations for the Care and use of Laboratory Animals of the National Institutes of Health. The protocol was approved by the University of Michigan Committee on Use and Care of Animals (University's Animal Welfare Assurance Number: A3114–01; Approval Number: 08905). All efforts were made to minimize suffering of the animals.

### 2. Verification of trait differences in inherent running capacity at week 11

The protocol for estimation of inherent running capacity required two weeks and was started when the rats were 11 weeks old. During the first week, each rat was initiated to a motorized treadmill (Model Exer-4, Columbus Instruments, Columbus, OH) over a 5-day period. The first two days of treadmill exposure consisted of simply placing each rat on the belt that was moving at a velocity of 10 meters per minute (m/min) (15° slope) and picking the rat up and moving it forward if it started to slide off the back of the belt. On days 3–5, the speed of the treadmill belt was gradually increased up to 15 m/min, and failure to run caused the rat to slide off the moving belt and into a 15 cm×15 cm electric shock grid for about 1.5 seconds and then moved forward onto the moving belt. This process was repeated until each rat learned to run to avoid the mild shock. The goal of the first week was to expose each rat to sufficient treadmill exposure until the animals were able to run 5 minutes at 10 m/min on a 15° slope. This amount of treadmill exposure is below that required to produce significant change in running capacity [20].

During the second week, each rat was tested once/day for 5 days at a constant slope of 15° and an initial velocity of 10 m/min. Treadmill velocity was increased by 1 m/min every 2 minutes until each rat had run to exhaustion. Exhaustion was operationally defined as the third time a rat could no longer keep pace with the speed of the treadmill belt and remained on an electric shock grid for 2 seconds rather than running. Extreme care was taken to ensure that HCR and LCR animals received the same amount of encouragement during running to exhaustion in the form of tapping on the treadmill or time spent on the shockers.

### 3. Treadmill tests and tissue collection

At 30 weeks of age (19 weeks after the initial test for verification of trait differences), each rat was re-tested on a treadmill as described above for varying durations of exercise. Eight LCR and 8 HCR rats were run to exhaustion (15.14±1.46 minutes and 51.23±1.85 minutes, respectively). In addition, two control groups were tested on the treadmills. In the first control group, eight LCR rats were run to 50% of LCR time to exhaustion (7.5 minutes). The purpose of the first control group was to include a group of LCR rats that were subject to acute treadmill exposure and would eliminate any handling confounds. In the second control group, eight HCR rats were time-matched to the LCR time to exhaustion (15 minutes). The purpose of the second HCR group was two-fold. First, this HCR group would serve as a time-matched control to LCR, with the onset of running and sacrifice time being matched. This matching would allow for direct comparison of c-Fos mRNA levels between HCR and LCR. Second, this HCR group would serve as an acute exercised control to HCR rats run to exhaustion. A non-exercise control group could not be included in this experiment since our output measure, c-Fos mRNA, is dependent on stimulus activation.

Thirty minutes post-treadmill running, when the accumulation of mRNA levels has reached its peak [81], all rats were sacrificed by rapid decapitation. Brains were removed and flash-frozen in cooled isopentene (−40 to −50°C). Trunk blood was obtained from the rats to determine their plasma corticosterone levels. A 0.5 ml sample of blood was collected in a microcentrifuge tube within 2–3 minutes. Once clotted, the blood was centrifuged, and the plasma was extracted and stored at −80°C. Both brains and blood were then shipped to the University of Colorado at Boulder for further processing.

### 4. In situ hybridization

Brain slices (10 µm thick) were thaw-mounted directly onto polylysine-coated slides and stored at –80°C until processing for single-labeled radioactive *in situ* hybridization as described previously [82], [83]. Briefly, sections were fixed in 4% paraformaldehyde for 1 hr, acetylated in 0.1 M triethanolamine containing 0.25% acetic anhydride (10 min), and dehydrated in graded alcohol. Riboprobes of cRNA (courtesy of Dr. T. Curran, St. Jude Children's Research Hospital, Memphis, TN) complementary to c-Fos (680 bp) were prepared from cDNA subclones in transcription vectors and labeled with [S-35]UTP (Amersham Biosciences, Piscataway, NJ), using standard transcription methods. Riboprobes were diluted in 50% hybridization buffer containing 50% formamide, 10% dextran sulfate, 2X saline sodium citrate (SSC), 50 mM PBS at pH 7.4, 1X Denhardt's solution, and 0.1 mg/ml yeast tRNA. Brain sections were hybridized with the probe overnight (55°C). The next day, sections were washed in 2X SSC, treated with RNase A (200 µg/ml) for 1 hour at 37°C, and washed to a final stringency of 0.1X SSC at 65°C for 1 hour. Dehydrated, air-dried sections were exposed to X-ray film (Biomax-MR; Eastman Kodak, Rochester, NY) for 1 week. Slides (each containing 4 brain sections) from all rats exposed were processed in a single experiment to allow for direct comparisons. Control experiments with “sense” probes indicated that the labeling observed with the “antisense” probes was specific (data not shown).

### 5. Image analysis

Computer-assisted optical densitometry was used to analyze c-Fos mRNA levels. Brain section images were captured digitally (CCD camera, model XC-77; Sony, Tokyo, Japan), and the relative optical density of the x-ray film was determined using Scion Image Version 4.0. A macro was written that enabled signal above background to be determined automatically. For each section, a background sample was taken over an area of white matter, and the signal threshold was calculated as mean gray value of background +3.5 standard deviations. The section was automatically density-sliced at this value, so that only pixels with gray values above these criteria were included in the analysis. Results are expressed as mean integrated density, which reflects both the signal intensity and the number of pixels above assigned background (mean signal above background x number of pixels above background). Each subject's mean integrated density of a particular cRNA probe at a given level represents the average of three slices chosen for analysis. Care was taken to ensure that equivalent areas were analyzed between animals.

### 6. Corticosterone assay

Plasma was assayed for corticosterone with an Enzyme ImmunoAssay kit from Assay Design, Inc. (Cat#901–097, Ann Arbor, Michigan). All samples were diluted 1:50 and assayed per kit instructions.

### 7. Statistical analysis

Group differences in body weight, treadmill speed, time, distance, corticosterone, and c-Fos mRNA levels were analyzed with one-way analysis of variance (ANOVA) tests using the StatView data analysis and presentation system (Power PC Version 5.0.1, Cary, North Carolina, USA). Fisher protected least significant difference (F-PLSD) post hoc analysis was performed when required. Correlational analyses were performed between the levels of c-Fos mRNA and distance run during treadmill tests at week 30. In all analyses, an alpha level of p<0.05 was used to determine statistical significance.
